# Supplementary material for: Deletion of Mfsd2b impairs thrombotic functions of platelets
Source: Nat Commun. 2021 Apr 16;12:2286. doi: 10.1038/s41467-021-22642-x (PMC8052357; doi:10.1038/s41467-021-22642-x)
Supplement: Supplementary file 1 — Supplementary Information [file 41467_2021_22642_MOESM1_ESM.pdf]

SUPPLEMENTARY INFORMATION

Deletion of Mfsd2b impairs thrombotic functions of platelets

This file includes:

Supplementary Figures 1-10.

Supplementary Methods.

Supplementary References.

Supplementary Figure 1.

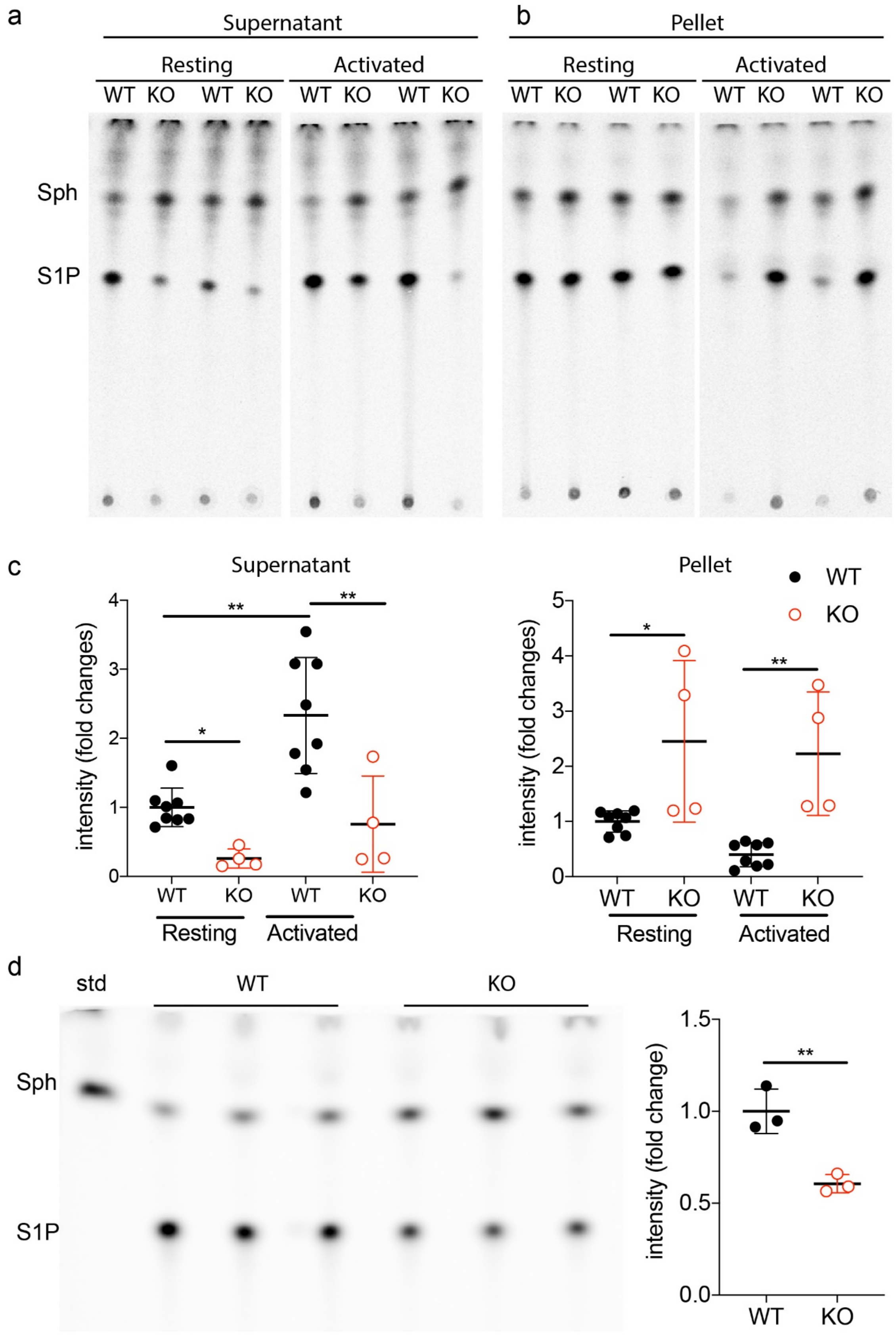

Supplementary Figure 1. Mfsd2b is required for S1P transport in platelets. **a-b**, representative images of TLC analysis from S1P transport assays using resting and activated platelets from WT and KO. The Sph and S1P bands were confirmed by Sph and S1P standards (see uncut gels). S1P in supernatant (a) and in cell pellets (b) were analyzed. WT: wild-type; KO: knockout; Sph: sphingosine; S1P: sphingosine-1-phosphate. **c**, Quantification of S1P band from those experiments as described in **a** and **b**. Data were analyzed from 2 different experiments performed with 8 WT and 4 KO. Data are mean and SD. *$P<0.05$, **$P<0.01$. One-way ANOVA was used. **d**, S1P transport assay using NBD-sphingosine as substrate with thrombin-activated platelets. Each dot represents one mouse (n=3). Data are mean and SD. **$P<0.01$. Two-tailed unpaired t-test was used. std: standard.

Supplementary Figure 2.

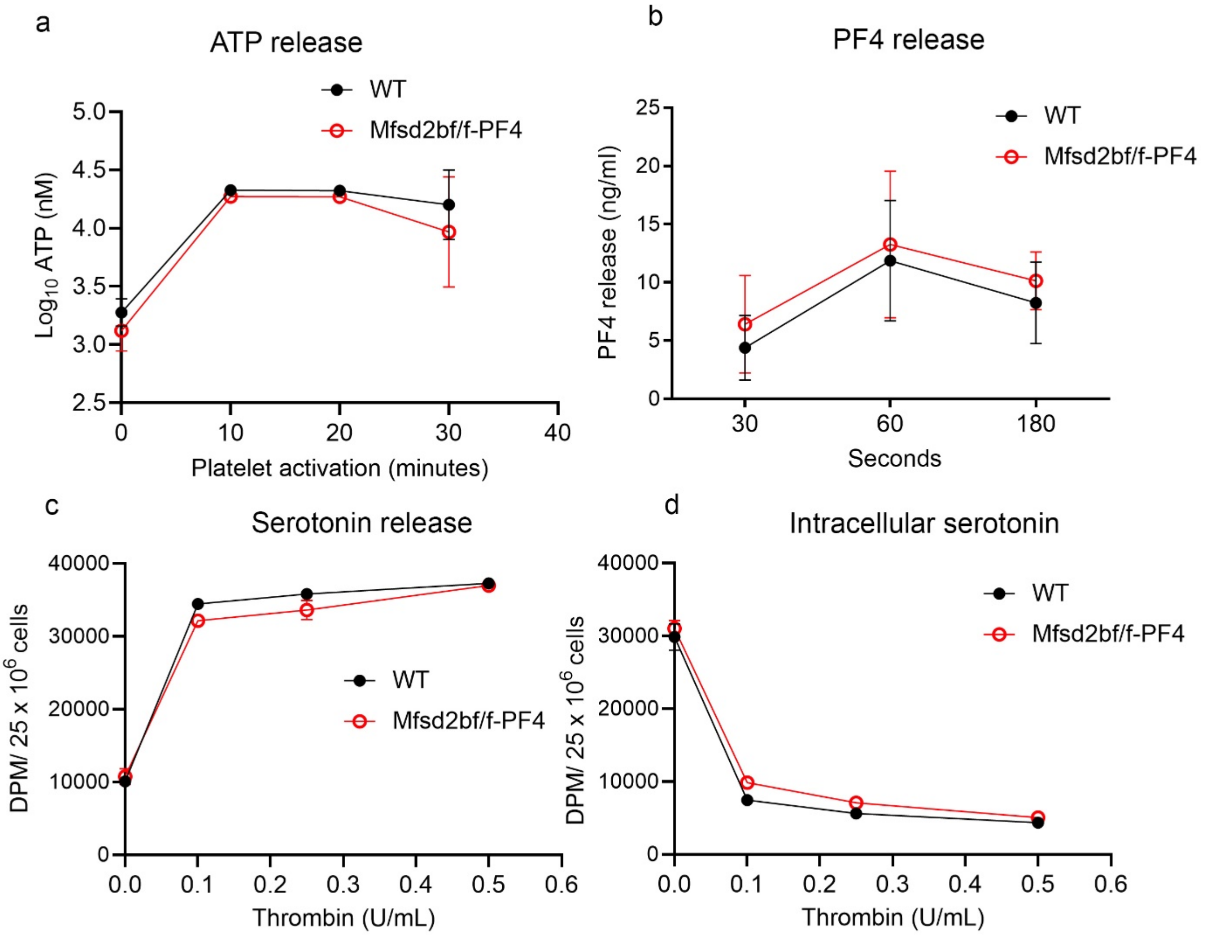

Supplementary Figure 2. Deletion of Mfsd2b does not affect the release of granular contents. a, Release of ATP from thrombin-activated platelets isolated from WT and Mfsd2bf/fPF4 knockout mice. ATP: adenosine triphosphate; WT: wild-type. **b,** Release of PF4 from thrombin-activated platelets isolated from WT and Mfsd2bf/f PF4 knockout mice. **c-d**, Intracellular and extracellular levels of serotonin released from thrombin-activated platelets isolated from WT and Mfsd2bf/f PF4 knockout mice. [3H] serotonin was used. Experiments were performed twice. n=8 per genotype before activation and n=4 after activation in **a**; n=4 in **b**; n=3 in **c** and **d**. Data are presented as mean and SD. DPM: disintegrations per minute.

Supplementary Figure 3.

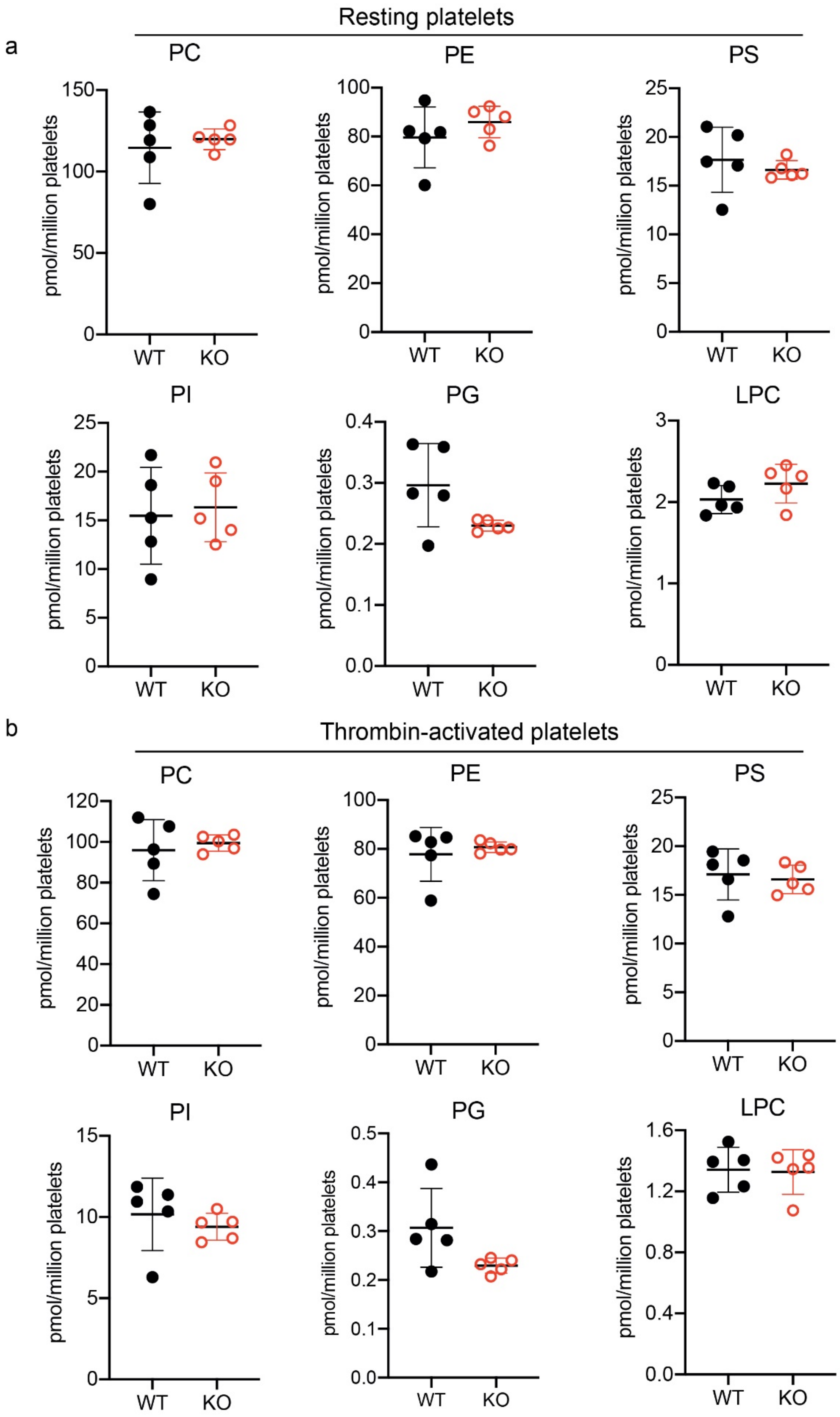

Supplementary Figure 3. Deletion of Mfsd2b did not change phospholipid levels in resting and activated platelets. Lipidomic analysis of major phospholipids in resting (**a**) and in activated (**b**) platelets from WT and KO mice. Shown values are total concentrations of different phospholipid classes (sum of all measured molecular species in each class) found in resting and activated platelets. Phosphatidylcholines (PC), phosphatidylethanolamines (PE), phosphatidylserines (PS), phosphatidylinositols (PI), phosphatidylglycerols (PG), and lysophosphatidylcholines (LPC). Data are mean and SD. Each dot represents one mouse (n=5). Individual lipid species can be found in the supplementary data 1.

Supplementary Figure 4.

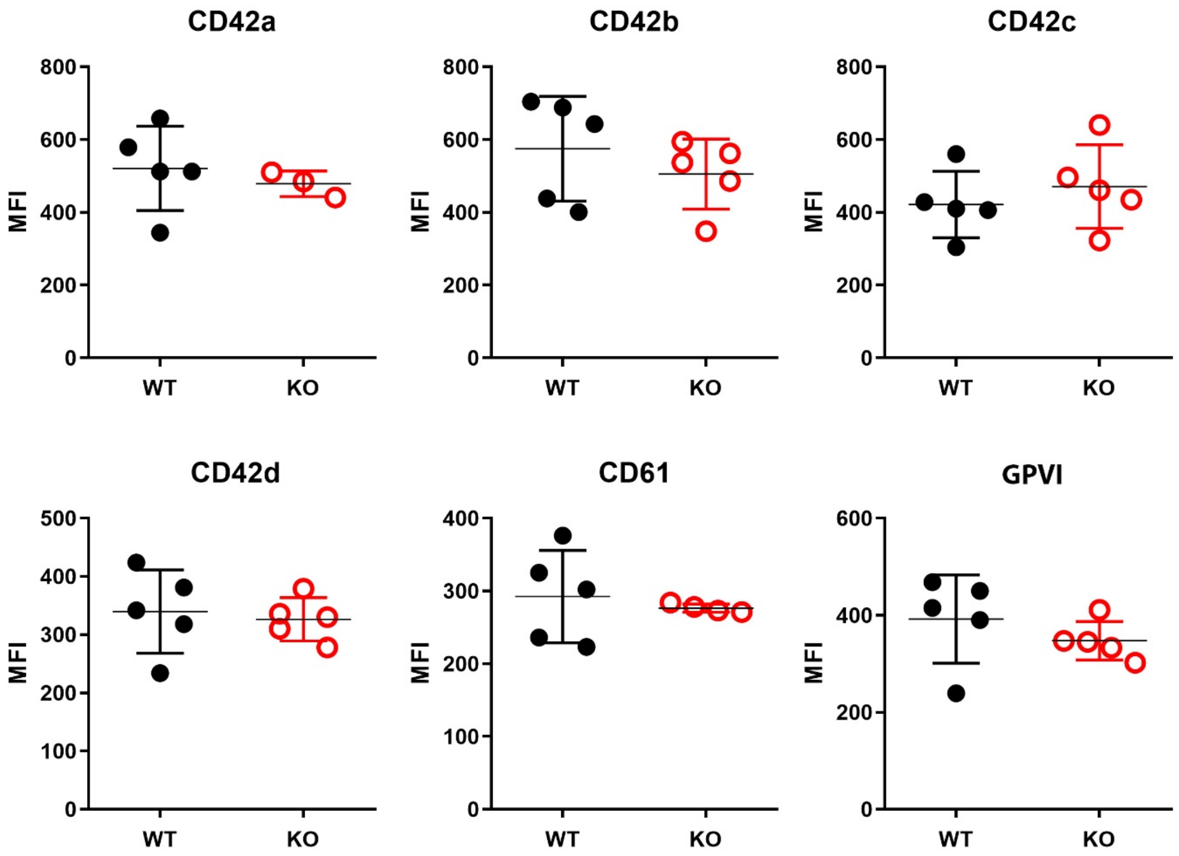

Supplementary Figure 4. Normal expression level of surface markers on Mfsd2b KO platelets. Flow cytometry assays for expression of indicated surface markers in whole blood isolated from WT and Mfsd2b KO mice. At resting states, Mfsd2b KO platelets exhibited normal expression of indicated surface markers. Representative data from two independent experiments. Each dot represents one mouse (n=3-5). Data are mean and SD. MFI: mean fluorescence intensity. See supplementary figure 10 for gating strategy.

Supplementary Figure 5.

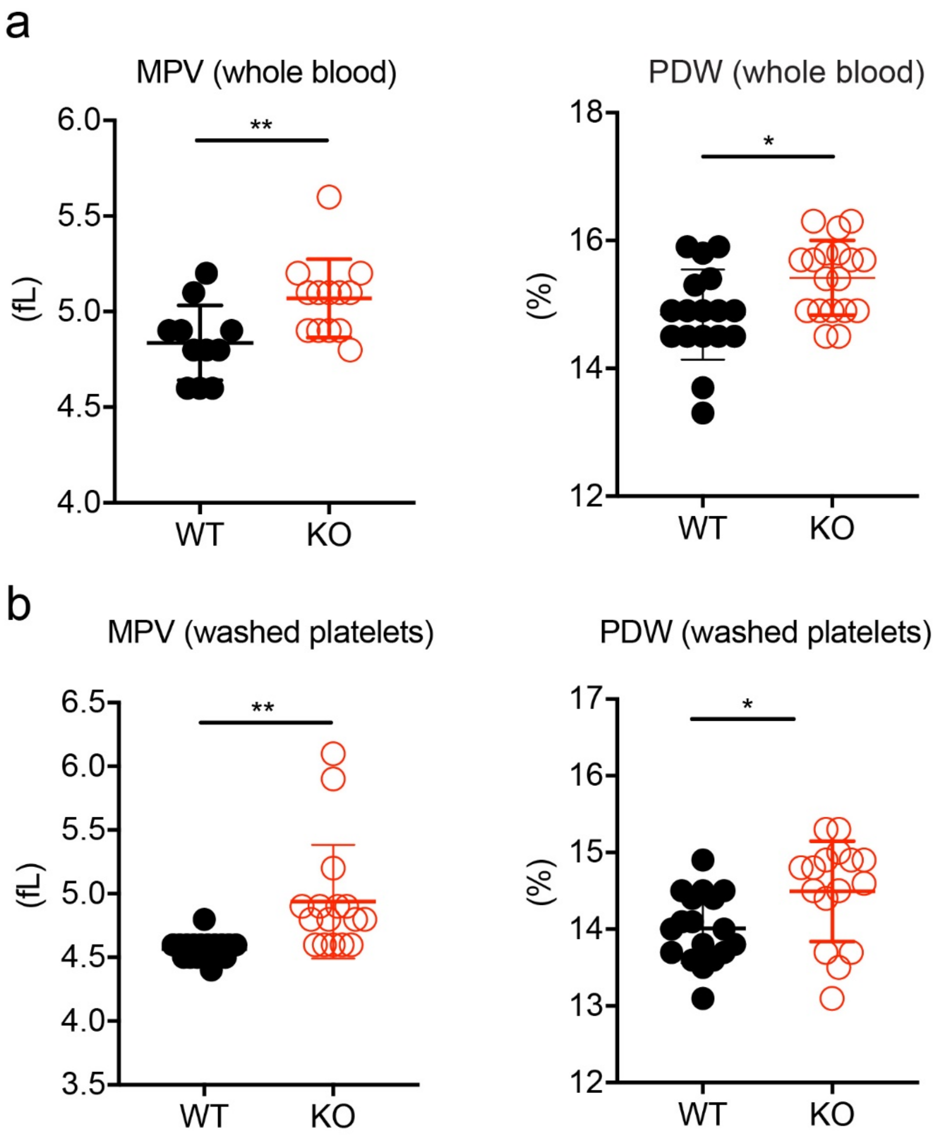

Supplementary Figure 5. Deletion of Mfsd2b resulted in altered platelet volume. Mean platelet volume (MPV) and platelet distribution width (PDW) from whole blood (**a**) and washed platelets (**b**) of WT and KO mice. Data are mean and SD. Each dot represents one mouse (n=11-13 for for MPV and n=17-18 for PDW in **a**; n=16-18 in **b**. *P<0.05, **P<0.01. Two tailed unpaired t-test was used. fL, femtoliter.

Supplementary Figure 6.

Activated WT (calcium ionophore A23187, 5 mins)

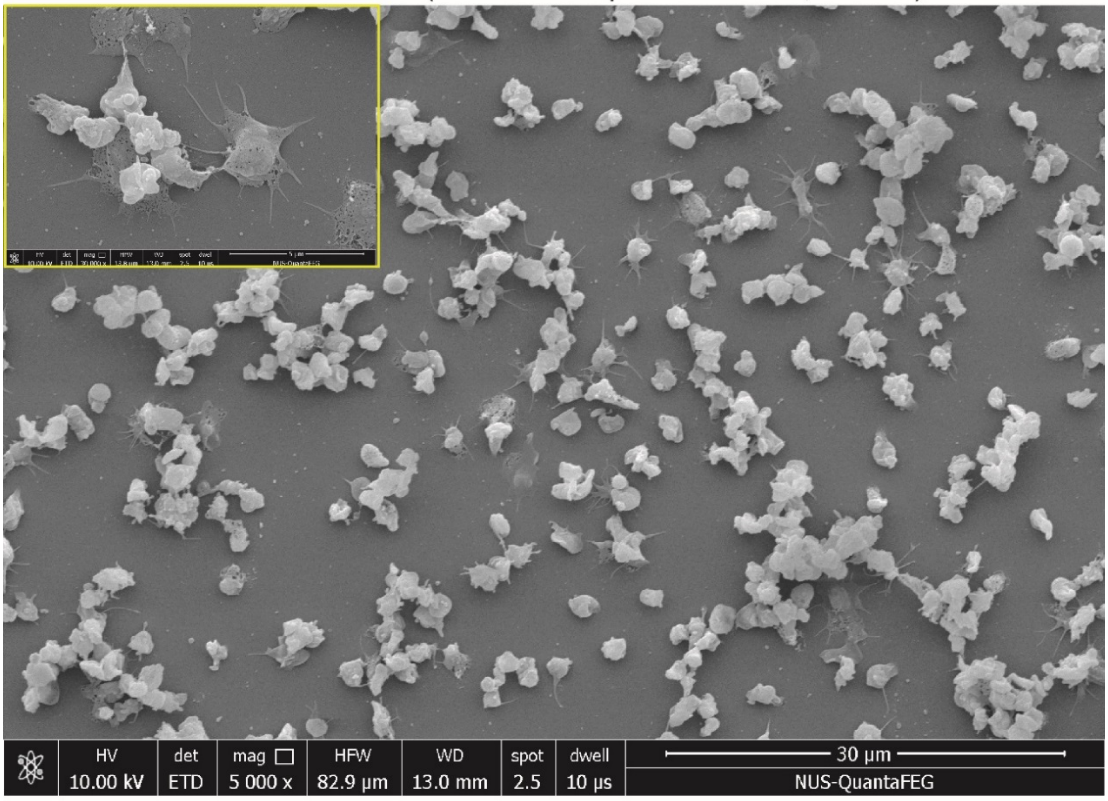

Activated KO (calcium ionophore A23187, 5 mins)

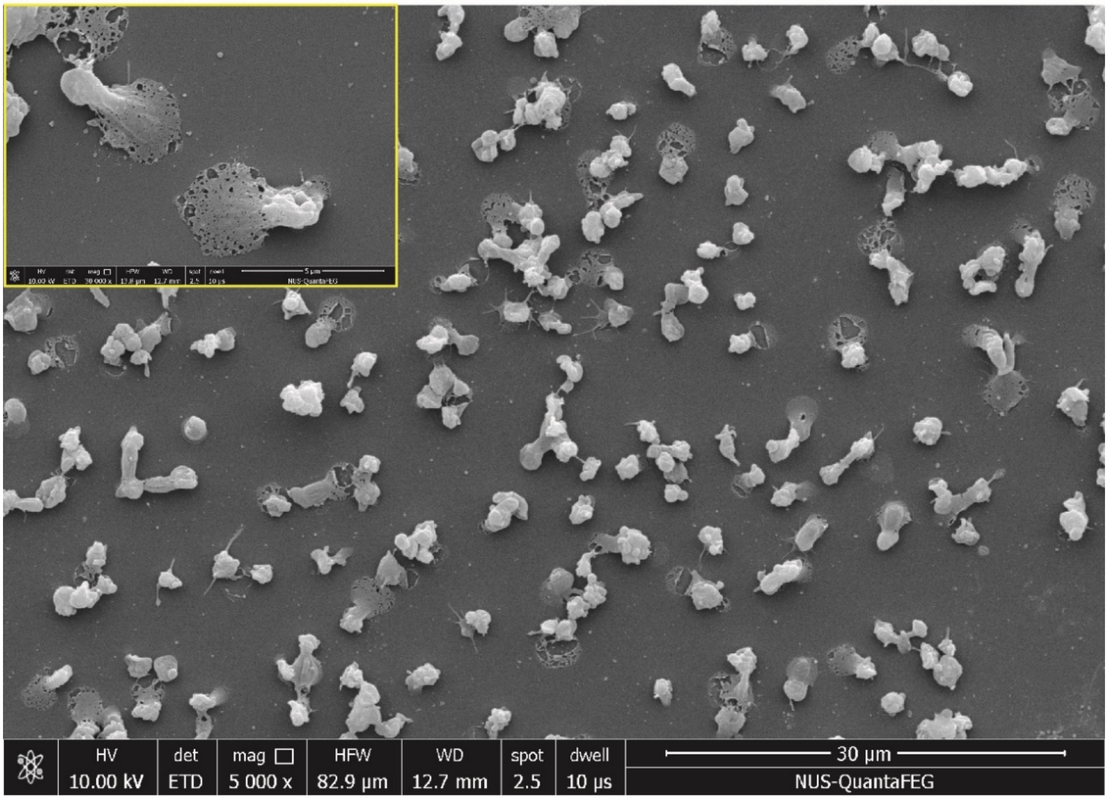

Supplementary Figure 6. Mfsd2b deficient platelets showed abnormal morphology after activation. Representative images of scanning electron microscopic (SEM) images of calcium ionophore A23187-activated WT and KO platelets. Platelets from Mfsd2b knockout mice exhibited reduced filopodia, but increased lamellipodia spreading after activation. Experiments were performed twice (n=2-3).

Supplementary Figure 7.

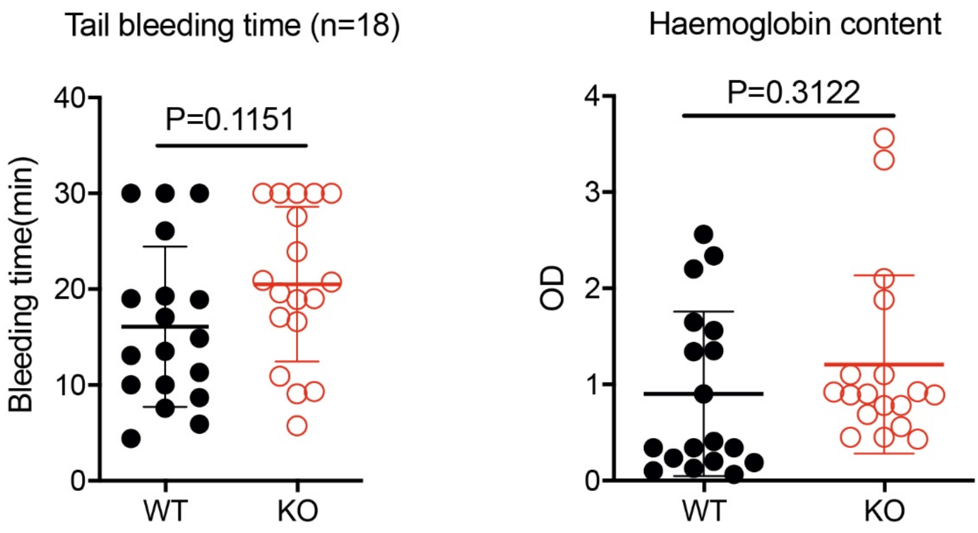

Supplementary Figure 7. Deletion of Mfsd2b did not significantly result in increased tail vein bleeding. Tail vein bleeding was comparable between WT and KO mice as indicated by bleeding time and amount of haemoglobin release. Although we noted that bleeding of Mfsd2b knockout mice was slightly stronger when performed orbital vein bleeding, we did not observe a significantly greater bleeding in the knockout mice with tail vein bleeding assay. Thus, we noted the limitations of this assay to measure bleeding in mice. Data are presented as mean and SD. P values were calculated using two-tailed unpaired t-test. WT: wild-type; KO: knockout; OD: optical density.

Supplementary Figure 8.

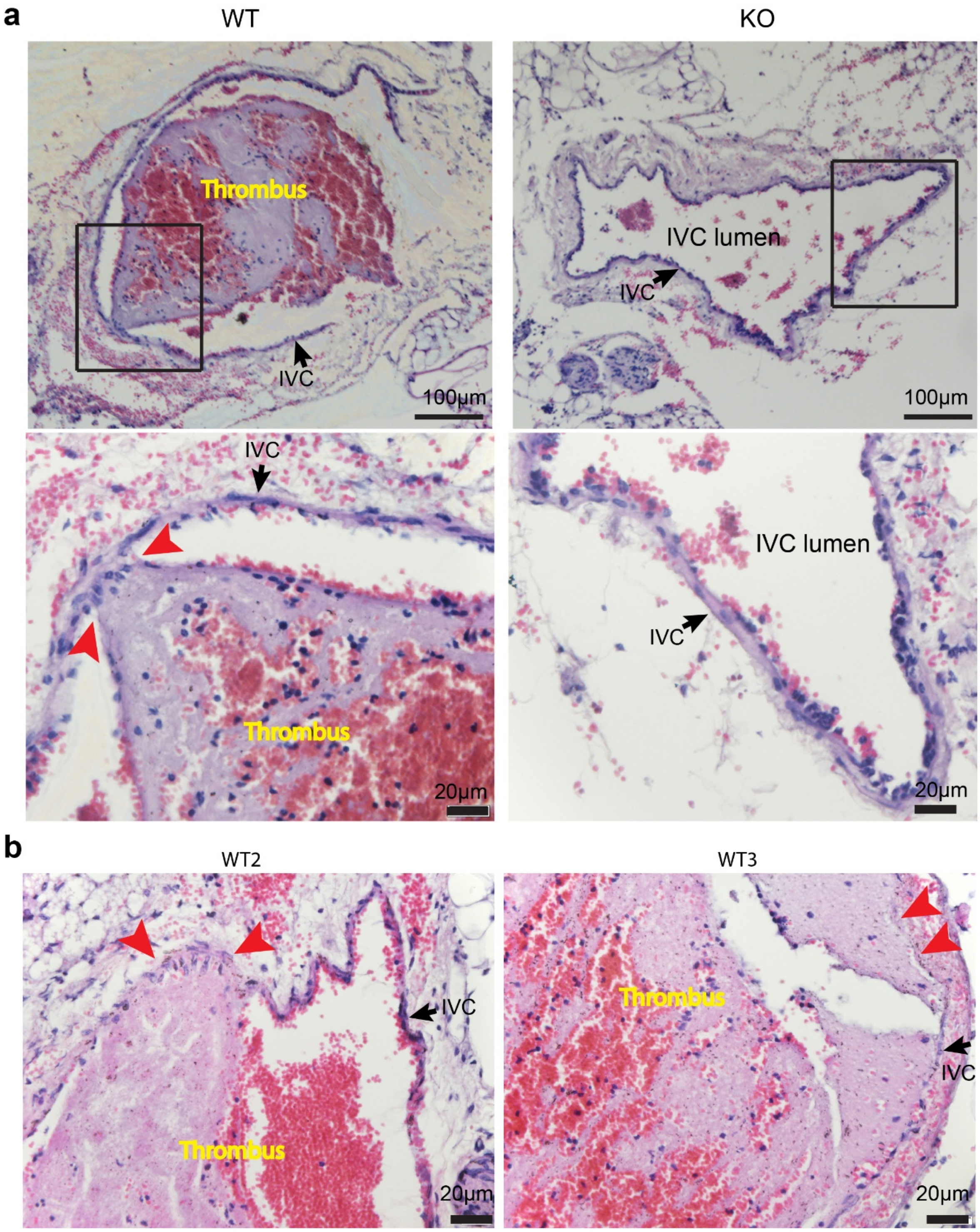

Supplementary Figure 8. Thrombosis was strongly reduced in Mfsd2b knockout mice. a, Histological assessment of IVC after 6 hours of stenosis. Venous thrombosis formation was found in WT mice, but not in KO mice after 6h of DVT. There were only small blood clots in IVC lumen from KO mice. We noted that there was likely attachments of white pulp to IVC from thrombus of WT mice as shown by the arrowheads. **b**, Similar results as described in a were observed in IVC isolated from different WT mice. WT: wild-type; KO: knockout; IVC: inferior vena cava. Experiments were performed at least twice (n=2-3).

Supplementary Figure 9.

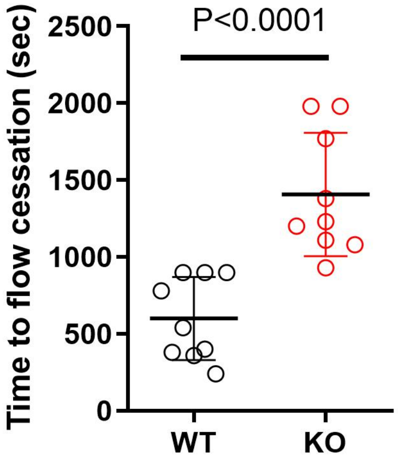

Supplementary Figure 9. Deletion of Mfsd2b resulted in reduced arterial thrombosis. Carotid arterial thrombosis induced by FeCl3. Global Mfsd2b KO mice had a longer time for blood occlusion compared with WT mice. Each dot represents one mouse (n=9). Data are mean and SD. Two-tailed unpaired t-test was used. WT: wild-type; KO: knockout.

Supplementary figure 10

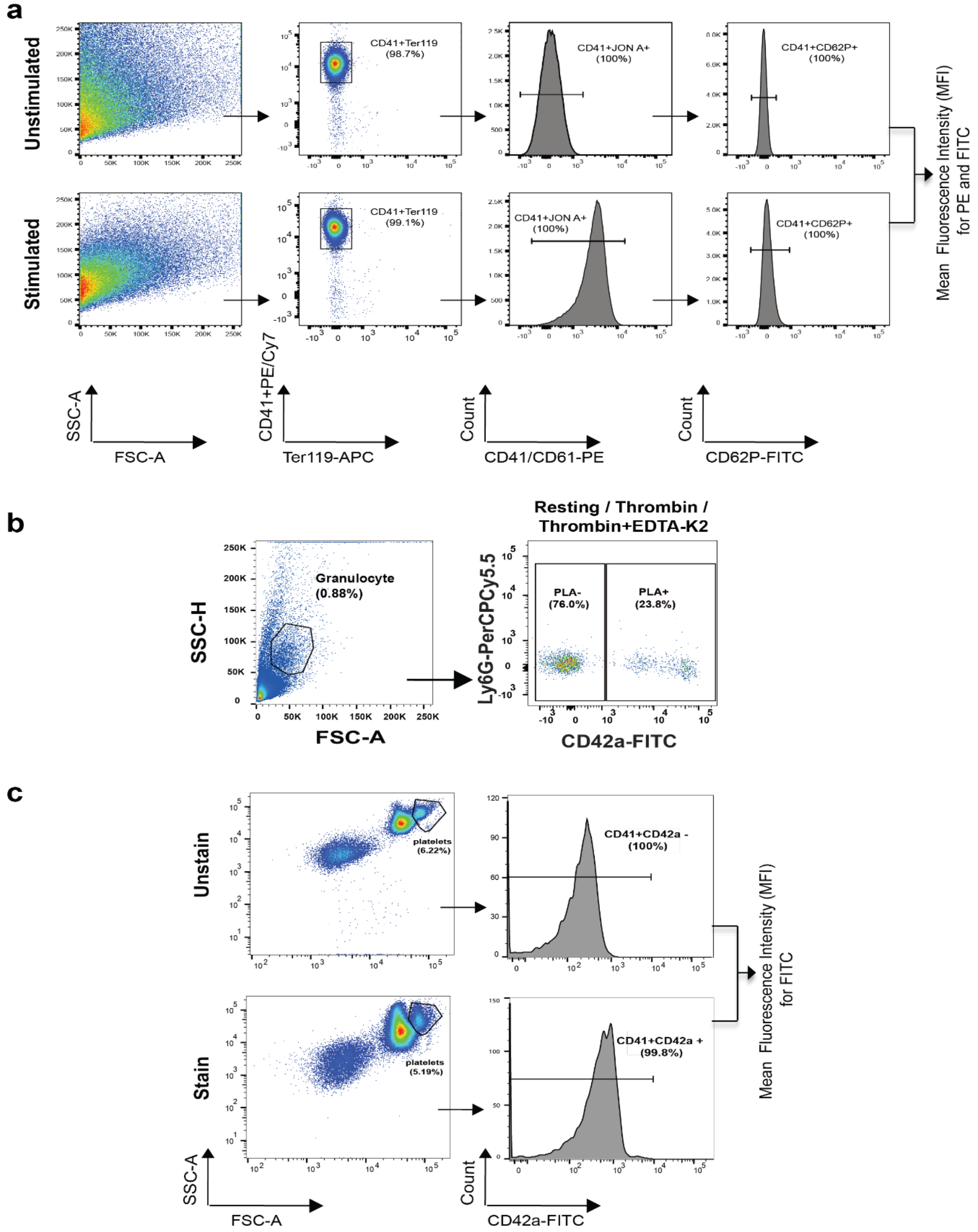

Supplementary figure 10. Gating strategies for flow cytometry. a, Gating strategy for JONA (CD41/61) and P-selectin (CD62P) expression on platelets to figure 5c-d. **b**, Gating strategy for platelet-neutrophil aggregation (PLA) assay for figure 8g. **c,** Gating strategy for the expression level of various surface markers of platelets for supplementary figure 4.

Supplementary Methods

Chemicals. Sphingosine (Sph, D-erythro-sphingosine) and sphingosine-1-phosphate (D-erythro-sphingosine-1-phosphate) were purchased from Avanti. Radiolabeled [3-^{3}H]sphingosine ([3-^{3}H]Sph) (stock specific activity: 1 μCi/μL) was purchased from American Radiochemicals. Fatty acid-free bovine serum albumin (BSA) was purchased from Sigma. All other reagents were purchased from Sigma. Lipids were solubilized in 12%BSA (free fatty acids BSA) in 150 mM NaCl. For preloading of S1P, 1 mM stock [3-^{3}H]sphingosine substrate dissolved in ethanol was used. Specific activity of [3-^{3}H]sphingosine in 1 mM Sph containing 12%BSA was 0.01 μCi/μL. For lipidomic analysis, internal standard mixture was prepared by combining the following standards in 1-butanol:methanol (1:1) 0.18 μM ceramide 17:0 (d18:1/17:0), 1.06 μM 12:0 sphingomyelin (SM, d18:1/17:0), 0.08 μM sphingosine-d7, 0.08 μM sphinganine-d7, 0.13 μM 12:0-13:0 phosphatidylinositol (PI), 0.18 μM 20:0 lysophosphatidylcholine (LPC), 0.70 μM 14:0 phosphatidylserine (PS), 0.07 μM 14:0 phosphatidylglycerol (PG), 2.89 μM 14:0 phosphatidylcholine (DMPC), and 0.77 μM 14:0 phosphatidylcholine (PE) that were purchased from Avanti Polar Lipids and 0.05 μM D-erythro-Sphingosine-1-phosphate-$^{13}C_2,D_2$ was purchased from Toronto Research Chemicals.

S1P release assay. Resting platelets and thrombin-activated platelets were incubated with [3-^{3}H]Sph (SA, 10μCi/μmol) in Tyrode H buffer for 30 min at 37°C to prepack the cells with [3-^{3}H]S1P. Cells were washed by Tyrode H buffer to remove excessive [3-^{3}H]Sph from medium before Tyrode H containing 0.5% BSA was added to facilitate the S1P release from the cells for 30 min at 37°C. In some transport assays, the conditions were mentioned in the figure legends. All centrifugation steps were performed at room temperature, 400 x g, using a swing rotor for 15 min without brake to minimize further platelet activation. Cell pellets and supernatant were separated by centrifugation and subjected to S1P extraction as previously described[1]. Levels of radioactive S1P were quantified by scintillation counter (Beckmann/Perkin Elmer Liquid scintillation counter).

Antibody. Rabbit polyclonal antibodies for Mfsd2b were generated as described previously [5] and used at 1:500 for Western blot. Western blots were re-probed with Anti-β actin (Sigma) or Gapdh antibody for loading control. P-selectin (CTB201: sc-8419, Santa Cruz), Na+/K+ ATPase (3010S, Cell Signaling), PF-4 (G-7: sc-374195, Santa Cruz). Podoplanin was purchased from eBioscience (clone 8.1.1). Antibodies for flow cytometry anti-CD42a (GPIX, Xia.B4, M051-1), anti-CD42b (GPIb, Xia.G5, M040-1), anti-CD42c (GPIbβ, Xia.C3, M050-1), anti-CD42d (GPIbα, Gon.C2, M060-1), anti-CD61 (GPIIIa, integrin β3, Luc.H11-M031-1), anti-GPIIbIIIa JON/A-PE - M023-2, anti-GPVI (JAQ1)-M011-1, anti-CD62P-FITC (M130-1) were obtained from Emfret analytics, Germany; and used at a concentration recommended by the manufacturer.

Western blot analysis. Washed platelets and washed erythrocytes were lysed by RIPA buffer (25 mM Tris pH 7–8, 150 mM NaCl, 0.1% SDS, 0.5% sodium deoxycholate 0.5% Triton X-100) supplemented with protease inhibitor cocktail (Roche). Platelet lysates of 30-50 µg were prepared in Laemmli loading buffer, resolved on 10% SDS-PAGE and transferred to nitrocellulose membrane. LiCOR-IR based system was used to detect and quantify the proteins of interest.

Platelet preparation for aggregation and S1P transport assays. Blood was collected from mouse by cardiac puncture in ACD-C solution (41.6 mM citric acid, 85.3 mM Trisodium-citrate, 136nM D-Glucose). Blood was diluted in 2:1 modified Tyrode H buffer (10 mM HEPES-NaOH,12 mM $NaHCO_3$, 138 mM NaCl, 5.5 mM D-glucose, 2.9 mM KCl, 1 mM $MgCl_2$, 0.3 µM PGE1 and 15% ACD) and centrifuged at 100 x g for 15 min to obtain the platelet rich plasma (PRP). This procedure was repeated again to obtain good platelet yield. PRP fraction obtained was centrifuged at 800 x g for 10 min. The platelet pellet was resuspended in Tyrode H buffer and counted using Celltac-α automatic analyser (MEK-6510, Nihon kohden, Japan).

Measurement of S1P release using thin layer chromatography (TLC). Washed platelets were prepared and resuspended at a density of 20 x 10^6 cells in Tyrode H buffer (pH 7.4) and incubated with [3-^{3}H]-Sph in a final concentration of 10 µM for 1 h at 37°C. Platelets were stimulated by 0.1 U/mL thrombin for 1 h and followed by a centrifugation to collect supernatant and cell pellets. Lipids extraction was performed using organic solvents from supernatant and cell pellet and spotted on precoated silica

plates. TLC plates were scanner by Storm scanner after exposure to a tritium phosphorscreen for 3 days.

Time course S1P transport assay. Briefly, 20 million platelets was isolated from WT and Mfsd2b KO mice whole blood and activated by 0.1 U/mL thrombin for 30 min before used for time course transport assay. Cells were washed once after activation with Tyrode H buffer and incubated with 2 µM of [3-^{3}H] Sph in 200 µL of Tyrode H containing 0.5% BSA at 37°C and reaction was stopped at 10, 20, 45, 60, and 90 min by centrifugation at 800 x g for 10 min at 4°C. Cells and supernatant were subjected to S1P extraction and quantified by scintillation counter.

Whole blood aggregation and flow cytometry. Platelet aggregation in whole blood was measured using multiple electrode aggregometry in multiplate analyser (Roche diagnostics, USA). Briefly, whole blood from WT, global Mfsd2b knockout, platelet specific knockout of Mfsd2b (Mfsd2bf/fPF4cre) and Mfsd2bf/f mice was collected in Hirudin **(**Multiplate® Hirudin Blood Tube) and incubated with 0.9% NaCl for 2 min at 37°C and followed by an induction with different agonists as follows: ADP (2 and 8µM final concentration) or calcium ionophore A23187 (2 µM) in the presence of 1 mM $CaCl_2$, Collagen (10 µg/mL), PAR-4 (45 µM). Aggregation was measured as the parameter area under the aggregation curve (AU).

Washed platelet aggregation. Washed Platelets (3.5x10^{8}/mL) from global WT and Mfsd2b KO mice were pre-incubated with 0.9% NaCl for 2 min at 37°C. Platelet aggregation was induced by ADP (8 µM) and A23187 (1 µM). Fibrinogen (5 µg/mL) was added prior to pre-incubation with the agonists. Aggregation was measured as area under the aggregation curve (AU).

Flow cytometry: For assessment of surface markers, whole blood diluted (1:20) in PBS+3%FCS (20 µL) stained with either 5 µL FITC conjugated-CD42a, CD42b, CD42c, CD42d, CD61, and GPVI antibodies and PE-CY7 CD41 (1 µL) for 15 min in dark and analysed immediately after adding 400 µL of PBS+3% FBS buffer. For analysis of αIIb3 activation and P-selectin exposure, integrin αIIb3 activation (binding of JON/A-PE) and degranulation dependent P-selectin exposure (FITC) was measured in response to platelet activation. Washed platelets from global WT and Mfsd2b KO mice (1 million cells) in Tyrode-H were stimulated with 1 µM A23187 in the

presence of 1 mM $CaCl_2$, for 15 min at 20°C and washed once with Tyrode-H buffer. The cells were stained with 5 µL JON/A -PE and 5 µL P-selectin FITC for 15 min. Then, 400 µL PBS+3% FBS buffer was added and analyzed immediately on a BD LSRFortessa. Results are expressed as mean fluorescent intensity (MFI) for positive PE or FITC of 3-5 mice per group.

Collection of blood, plasma, RBC and platelets and lipidomic analysis. Blood was collected from retro-orbital vein under isoflurane anesthesia. For plasma collection, peripheral blood was collected via orbital vein by heparin-collected capillary into EDTA-K2 collection tube. Plasma was separated from blood cells by centrifugation at 1258 x g at room temperature for 30 min. Plasma samples were kept at -80°C until further analysis. For RBC preparation, blood was collected retro-orbitally by heparin-collected capillary into EDTA-K2 collection tube and washed thoroughly at 4°C in PBS supplemented with 0.5% BSA and counted. Platelets were collected from peripheral blood in to ACD/Tyrode H (pH 7.4) buffer and centrifuged to collect the platelets and washed carefully to remove the RBC's as mentioned previously [1]. Platelets were activated with 1 U/mL thrombin. Lipidomic analyses were performed using liquid chromatography tandem mass spectrometry (LC-MSMS) on a UHPLC 1290 Agilent system connected to a 6495 QQQ Agilent mass spectrometer. Platelets and plasma samples were first spiked with internal standards and subjected to lipid extraction using 1-butanol/methanol (1:1) mixture. Major phospholipids from platelets as indicated in the text were measured as described previously[2]. Briefly, separation of the different classes was achieved using a Kinetex HILIC stationary phase (150×2.1 mm, 2.6 µm, 100 Å; Phenomenex). Gradient elution was performed with solvents A (50% acetonitrile + 50% 25 mM ammonium formate buffer at pH 4.6) and B (95% acetonitrile + 5% 25 mM ammonium formate buffer at pH 4.6), with a gradient starting at 99.9% B, to 25% solvent B in 6 min, to 10% solvent B in 1 min, back to 99.9% solvent B in 0.1 min, held at 99.9% solvent B for 3 min (total run time of 10.1 min). The flow rate was 0.5 mL/min. Phospholipids were quantified using multiple reaction monitoring.

Measurements of sphingomyelin, ceramide, sphingosine, and sphingosine 1-phosphate species were performed as described previously [3], [4]. For sphingolipids, reversed phase separation was performed using the Agilent Zorbax RRHD Eclipse

Plus C18 (100x2.1 mm, 1.8 µm) column. The flow rate was 0.4 mL/min and the column temperature was maintained at 40°C. Gradient elution was performed with solvents A (60% Methanol, 40% water, 0.2% formic acid, 10 mM ammonium acetate) and B (60% Methanol, 40% Isopropanol, 0.2% formic acid, 10 mM ammonium acetate) with a gradient range from 0% B to 10% B in 3.0 min, 40% B at 5.0 min, 55% B at 5.30 min, 60% B at 8.0 min, held at 80% B from 8.50 min to 10.5 min, held at 90% B from 16.0 min to 19.0 min, 100% B at 22.0 min and re-equilibrated at 0% B from 22.1 min to 25.0 min. The sphingolipid levels were quantified using dynamic multiple reaction monitoring method (dMRM).

For S1P analysis, derivatization of S1P was carried out by adding 10 µL of TMS-diazomethane to 100 µL of the lipid extract and shaking on thermomixer at 700 rpm for 20 min at 23°C. After 20 min, 1 µL of 100% acetic acid was added to stop the derivatization reaction. Samples were vortexed, then centrifuged at 16,000 x g for 10 min to remove potential debris before transferring into a 96-well plate. Plates were stored at 4°C until LC-MS/MS analyses using an Agilent 1290 UPLC system connected to an Agilent 6495 Triple Quadrupole mass spectrometer operated in positive ion MRM mode. The column utilized was an Acquity hydrophilic interaction chromatography (HILIC) column (100×2.1 mm, 1.7 µm particle size, Waters Corporation). MS source parameters used: gas temperature of 200°C with gas flow of 12 L/min and nebulizer at 25 psi. Sheath gas temperature of 400°C with gas flow of 12 L/min. Gradient elution was performed with solvents A (50% acetonitrile + 50% 25 mM ammonium formate buffer at pH 4.6) and B (95% acetonitrile + 5% 25 mM ammonium formate buffer at pH 4.6), with the following gradient: 0.1% solvent A and 99.9% solvent B from 0 to 5 min, 60% solvent A and 40% solvent B from 5 to 5.5 min, 90% solvent A and 10% solvent B from 5.5 to 6.6 min, 0.1% solvent A, and 99.9% solvent B from 6.6 to 9 min with a constant flow rate of 0.4 mL/min. After collision induced dissociation of the S1P precursors, two product ions were produced and monitored: *m/z* 60 was used as a "quantifier" and *m/z* 113 was used as the "qualifier." These two ions were present after fragmentation of all S1P molecular species. The ion with *m/z* 60 represents the trimethylated amine fragment while the ion with *m/z* 113 represents the mono-methylated phosphate as previously described.

Peak integration was performed using MassHunter Quantitative Analysis (QQQ) software, and data were manually curated to ensure correct peak integration. Areas under the curve for the extracted ion chromatogram peaks for each multiple reaction monitoring transition and lipid species were normalized to the relative internal standard. Isotope correction was then done on all lipid species. One-point calibration was used to calculate the molar concentrations of the lipids. For platelets, the data were further normalized to the number of platelets. The stability of signal throughout the analysis was monitored by regular injection of a quality control sample.

Isolation of platelets plasma membrane protein by biotinylation. An equal amount of 300 millions of resting platelets and thrombin-activated platelets were incubated with PBS/Ca2+/Mg2+ containing 0.5 mg/mL EZ Link Sulfo-NHS-SS-Biotin (Pierce) for 15 min at 4°C. Free biotin was quenched and removed by washing the cells 3 times with cold 100 mM glycine. Cells were lysed in 200μl RIPA buffer supplemented with protease inhibitor cocktail (Roche). Biotinylated proteins were isolated by incubating the lysates with 50μl of 50% slurry NeutrAvidin Plus UltraLink Resin beads (Pierce) for 2 hours at 25°C. The beads were washed three times with cold RIPA buffer before treated with 50 μl of 5x Laemmli denature buffer at 65°C for 15 min in order to elute the samples. Biotinylated protein fractions and total protein lysates were analyzed by SDS-PAGE and Western blot with antibodies specific for Mfsd2b, Integrin b3, PF4 and beta-actin.

Subcellular fractionation. To fractionate the cytosolic and membrane fractions, 100-300 million resting platelets were broken by 5 cycles of freeze-thaw[5]. Unbroken platelets were removed by centrifugation for 5 min at 4000 x g at 4°C. The supernatants were subjected to ultracentrifuge at 100,000 x g for 1 hour. The cytosolic and membrane fractions were harvested for analysis.

Tail bleeding measurement. Bleeding time in mice was measured using the tail tip transection method. Mice was anesthetized with ketamine 75 mg/kg and xylazine 10 mg/kg and a 3 mm transection was performed in the tail tip. The tail was immediately immersed in 50 mL 0.9% NaCl maintained at 37°C. Tail bleeding was observed for at least 30 min to ensure the complete cessation of bleeding and the time of permanent

cessation of blood was recorded. Since there is a great variable result obtained from this assay, we performed this experiment at least 4 times.

Carotid artery thrombosis. WT and Mfsd2b knockout male mice (9–11 weeks old, 24–28 g) were anesthetized with ketamine (75 mg/kg) and xylazine (10 mg/kg) by intraperitoneal injection (i.p.). Following this, the right carotid artery was exposed using blunt dissection and a doppler flow probe (Model MA0.5VB, Transonic System Inc., Ithaca, NY, USA) connected to a perivascular flow module (TS420, Transonic System Inc., Ithaca, NY, USA) was then attached to the carotid artery to monitor blood flow. A 2x2 mm piece of filter paper (soaked in 5% $FeCl_3$ solution) was placed on the surface of the carotid artery for 3 min to initiate the thrombus formation [6]. Time-to-occlusion (TTO) is defined as the time taken for the blood flow to reach zero after the application of $FeCl_3$. Maximum measurement time was considered for 30 min after the application of $FeCl_3$. TTO was recorded as 30 min, if no occlusion occurred by this time.

Mesenteric artery thrombosis. Mesenteric thrombosis model was followed Li Wei's protocol with minor modification[6]. Briefly, mice were anesthetized and injected with 50 µL of Dylight488 antibody (Emfret) to label platelets in vivo. Mesenteric arteries were exposed and viewed under microscope. To induce arterial thrombosis, 1mm^2 filter paper saturated with freshly prepared 7.5% $FeCl_3$ was applied to the artery for 3 min. Then, thrombosis was recorded after 3 min of $FeCl_3$ application under 10X objective in an Olympus microscope equipped with fluorescence. The recording of thrombosis was stopped at 20 min for all mice.

Transmission electron microscopy (TEM). WT and Mfsd2b KO washed platelets as obtained by the same method for transport assays and lipidomics analysis were fixed in 2.5% glutaraldehyde in 0.1 M phosphate buffer (pH 7.4) for 60 min at 4°C. The samples were washed, fixed with 1% osmium tetroxide in 0.1 M phosphate buffer for 60 min at 4°C, dehydrated with a graded ethanol series, and then embedded in Epon (TAAB Laboratories, Aldermaston, Berkshire, United Kingdom). Ultrathin sections were prepared, stained with uranyl acetate and lead citrate, and then examined with a JEM1200EX transmission electron microscope (JEOL, Tokyo, Japan) at an accelerating voltage of 80 kV.

Scanning electron microscopy (SEM). Washed platelets were coated on cover slips for 1 hr and activated with calcium ionophore 1μM A23187 in Tyrode H buffer containing 1 mM $CaCl_2$ for 5 min. Excess Tyrode H buffer was added to stop the activation and cells were fixed with 2.5% glutaraldehyde for 1 h at room temperature. Slides were post-fixed with 1% osmium tetroxide for 1h and dehydrated using a serial ethanol concentration. Images were taken using a Fei Quanta650 scanning electron microscope.

Granular release assays. For ATP release assay, the extracellular ATP released from platelet granules was quantified using a modified luciferase assay. Briefly, washed platelets ($2x10^8$/mL) from WT and Mfsd2bf/fPF4 knockout mice were activated with thrombin (0.5 U/mL) for the indicated time (10, 20 and 30 min) at 37°C. The activation was stopped at each time point by the addition of hirudin (0.5 U/mL) for 10 min. The supernatant containing the released granular ATP was collected via centrifugation at 12,000 x g for 10 min. A volume of 25μl of supernatant was mixed with 25μl of luciferase/luciferin reagent (ENLITEN®, Promega, Cat. No. FF2000) and luminescence was immediately measured by a SpectraMaxM2 microplate reader. The ATP content was calculated using the values obtained from an ATP standard curve and expressed in Log ATP (nM) concentration. For serotonin release assay, 400 million platelets from WT and Mfsd2bf/fPF4 knockout mice were prepared as described above and were labelled with [3H]-serotonin (0.4 μCi/mL) in 500 μL Tyrode H for 1 hour at 37°C. Platelets were resuspended and adjusted to 2×10^8/mL and activated with 4 different concentrations of thrombin (0, 0.1, 0.25, and 0.5 U/mL, respectively) for 1 min. Activation was stopped by adding 2-fold excess of hirudin (1 U/mL). Radioactive signals from supernatants and cell pellets were quantified. For platelet factor 4 (PF4) release from α-granules, PF4 was measured by ELISA accordingly to manufacturer instructions (Abcam, ab202403). In brief, 2.5 $x10^6$ million washed platelets were activated with 0.1 U/mL thrombin in 50 μL of Tyrode-H buffer in the presence of 0.7 mM $CaCl_2$ for 30, 60 and 180 seconds. Reactions were stopped with 0.2 U/mL of hirudin (Sigma-Aldrich, 0393-100UN). Supernatant was collected after centrifugation at 13,000 g for 1 min at RT and used for assay. The OD was read at 450 nm in SpectraMax M2 plate reader.

Platelet-neutrophil aggregation assay. Washed PRP was prepared from peripheral blood in Tyrode-H buffer. Leukocytes were collected from the WT and Mfsd2b KO mice using in Tyrode-H buffer followed by centrifuge at 600 x g for 5 min at room temperature (RT). RBC was removed by adding RBC lysis buffer for 30 min at RT followed by washing with PBS twice and resuspended in 50 µL of Tyrode-H buffer and stained with Ly6G (Biolegend, 127616) for neutrophils of 15 min at RT. Finally, the volume of leukocyte samples was readjusted to 150 µL by Tyrode-H buffer and used as label neutrophils/granulocytes for aggregation assay with platelets collected from WT and global Mfsd2b KO mice. For labelling of platelets, an amount of 30 µL PRP from WT and global Mfsd2b KO mice and the Ly6G labelled neutrophils were added in the 1.5 mL micro centrifuge tube containing CD42a, $CaCl_2$ (1 mM) for resting condition, or thrombin (1 U/mL) activation condition, or thrombin (1 U/mL) plus EDTA-K2 (15 mM) condition. The samples were incubated for 30 min at 37°C for aggregation between platelets and neutrophils. Finally, the reactions were stopped and fixed with 1x BD FACS lysis buffer (BD, cat# 349202) for cytometry analysis. The double positive signals for platelets and neutrophils were detected by flow cytometry with 1000 events.

Transfusion of WT platelets into Mfsd2b mice. Transfusion experiments were performed by infusing 800 million WT platelets into WT and global Mfsd2b KO mice, and 800 million platelets from Mfsd2b KO to KO mice. Brief, blood was collected from the retro-orbital plexus of WT C57Bl/6 mice and global Mfsd2b KO mice in sodium citrate and centrifuged at 80g for 15 min to obtain platelet-rich plasma. Red blood cells were sedimented by centrifugation (80 x g; 5 min), and the supernatant (platelet-rich plasma) was incubated with prostaglandin I_2 (2 mg/mL, 5 min, 37°C) and centrifuged (1500 x g, 5 min). The platelet pellet was resuspended in modified Tyrode’s buffer. Platelets were pooled from a number of donor mice. After pooling, platelets (8×10^8 in 200 mL of buffer) were transfused into WT and Mfsd2b KO mice without prior platelet depletion. Immediately after transfusion, recipient mice were subjected to IVC stenosis, and thrombus formation was checked after 48 hours.

JTE-013 treatment. To prepare JTE-013 for gavage, JTE013 was first diluted in ethanol to 10-20 mg/mL. This solution is solubilized in corn oil and ethanol is blown off with N2 gas. The final JTE013 concentration in corn oil is 10 mg/mL. The mice were

gavaged with 30mg/kg. For DVT, mice were gavaged twice for 2 days, day 0 (before DVT) and day 1. Thrombus was collected after 48hrs post-surgery.

References in the method section

1. Vu TM*, et al.* Mfsd2b is essential for the sphingosine-1-phosphate export in erythrocytes and platelets. *Nature* **550**, 524-528 (2017).

2. Wong BH*, et al.* Mfsd2a Is a Transporter for the Essential omega-3 Fatty Acid Docosahexaenoic Acid (DHA) in Eye and Is Important for Photoreceptor Cell Development. *J Biol Chem* **291**, 10501-10514 (2016).

3. Burla B, Muralidharan S, Wenk MR, Torta F. Sphingolipid Analysis in Clinical Research. *Methods Mol Biol* **1730**, 135-162 (2018).

4. Narayanaswamy P*, et al.* Lipidomic "deep profiling": an enhanced workflow to reveal new molecular species of signaling lipids. *Anal Chem* **86**, 3043-3047 (2014).

5. Ye S*, et al.* Platelet secretion and hemostasis require syntaxin-binding protein STXBP5. *J Clin Invest* **124**, 4517-4528 (2014).

6. Li W, Nieman M, Sen Gupta A. Ferric Chloride-induced Murine Thrombosis Models. *J Vis Exp*, (2016).
