# Supplementary material for: Deletion of Mfsd2b impairs thrombotic functions of platelets
Source: Nat Commun. 2021 Apr 16;12:2286. doi: 10.1038/s41467-021-22642-x (PMC8052357; doi:10.1038/s41467-021-22642-x)
Supplement: Supplementary file 3 — Description of Additional Supplementary Files [file 41467_2021_22642_MOESM3_ESM.pdf]

Description of Additional Supplementary Files

File Name: Supplementary Data 1
Description: Raw and processed data for sphingolipids and phospholipids analysis by mass spectrometry.
