# Supplementary material for: Deletion of Mfsd2b impairs thrombotic functions of platelets
Source: Nat Commun. 2021 Apr 16;12:2286. doi: 10.1038/s41467-021-22642-x (PMC8052357; doi:10.1038/s41467-021-22642-x)
Supplement: Supplementary file 5 — Reporting Summary [file 41467_2021_22642_MOESM5_ESM.pdf]

nature research

Corresponding author(s): NCOMMS-20-05553C

Last updated by author(s): Feb 8, 2021

Reporting Summary

Nature Research wishes to improve the reproducibility of the work that we publish. This form provides structure for consistency and transparency in reporting. For further information on Nature Research policies, see Authors & Referees and the Editorial Policy Checklist.

Statistics

For all statistical analyses, confirm that the following items are present in the figure legend, table legend, main text, or Methods section.

n/a	Confirmed	
☐	☒	The exact sample size (*n*) for each experimental group/condition, given as a discrete number and unit of measurement
☐	☒	A statement on whether measurements were taken from distinct samples or whether the same sample was measured repeatedly
☐	☒	The statistical test(s) used AND whether they are one- or two-sided<br>*Only common tests should be described solely by name; describe more complex techniques in the Methods section.*
☐	☒	A description of all covariates tested
☐	☒	A description of any assumptions or corrections, such as tests of normality and adjustment for multiple comparisons
☐	☒	A full description of the statistical parameters including central tendency (e.g. means) or other basic estimates (e.g. regression coefficient) AND variation (e.g. standard deviation) or associated estimates of uncertainty (e.g. confidence intervals)
☒	☐	For null hypothesis testing, the test statistic (e.g. *F*, *t*, *r*) with confidence intervals, effect sizes, degrees of freedom and *P* value noted<br>*Give P values as exact values whenever suitable.*
☒	☐	For Bayesian analysis, information on the choice of priors and Markov chain Monte Carlo settings
☒	☐	For hierarchical and complex designs, identification of the appropriate level for tests and full reporting of outcomes
☒	☐	Estimates of effect sizes (e.g. Cohen's *d*, Pearson's *r*), indicating how they were calculated

Our web collection on statistics for biologists contains articles on many of the points above.

Software and code

Policy information about availability of computer code

Data collection	LiCOR-IR based system and ChemiDoc MP Imaging System for acquiring Western blot images. TRI-CARB Liquid Scintillation Counter for acquiring radioactive signals. Multiplate® Analyzer for acquiring aggregation parameters.
Data analysis	Microsoft excel, Graph pad prism, FlowJo

For manuscripts utilizing custom algorithms or software that are central to the research but not yet described in published literature, software must be made available to editors/reviewers. We strongly encourage code deposition in a community repository (e.g. GitHub). See the Nature Research guidelines for submitting code & software for further information.

Data

Policy information about availability of data

All manuscripts must include a data availability statement. This statement should provide the following information, where applicable:

- Accession codes, unique identifiers, or web links for publicly available datasets
- A list of figures that have associated raw data
- A description of any restrictions on data availability

Raw data will be provided upon request from corresponding author

Field-specific reporting

Please select the one below that is the best fit for your research. If you are not sure, read the appropriate sections before making your selection.

☒ Life sciences ☐ Behavioural & social sciences ☐ Ecological, evolutionary & environmental sciences

Life sciences study design

All studies must disclose on these points even when the disclosure is negative.

Sample size	Appropriate sample size was used to determine the statistic significance by ANOVA and t-tests. Sample size was determined from our previous publication (PMID: 29045386) and was stated in each figure legend.
Data exclusions	For platelet collection experiments where blood collection caused coagulation before platelets were collected for analysis, these samples were not included in analysis. There is no data that was excluded.
Replication	Experiments were repeated where possible. Experiments were repeated at least 2-3 times with at least 3 replicates.
Randomization	Samples and mice were randomized for DVT experimentation. Male and female mice were used where appropriate age-matched and gender-matched were applied. The DVT results have been reproduced from two different surgeons for this procedure.
Blinding	Deep vein thrombosis experiments were blinded where the surgeons were blinded for mouse genotype.

Reporting for specific materials, systems and methods

We require information from authors about some types of materials, experimental systems and methods used in many studies. Here, indicate whether each material, system or method listed is relevant to your study. If you are not sure if a list item applies to your research, read the appropriate section before selecting a response.

Materials & experimental systems

n/a	Involved in the study	
☐	☒	Antibodies
☒	☐	Eukaryotic cell lines
☒	☐	Palaeontology
☐	☒	Animals and other organisms
☒	☐	Human research participants
☒	☐	Clinical data

Methods

n/a	Involved in the study	
☒	☐	ChIP-seq
☐	☒	Flow cytometry
☒	☐	MRI-based neuroimaging

Antibodies

Antibodies used	-Mfsd2b were generated as described previously [PMID: 29045386] and used at 1:500 for Western blot. Western blots were re-probed with Anti-β actin (Sigma) or Gapdh antibody for loading control. P-selectin (CTB201: sc-8419, Santa Cruz), Na+/K+ ATPase (3010S, Cell Signaling), PF-4 (G-7: sc-374195, Santa Cruz). Podoplanin antibody was purchased from eBioscience (clone 8.1.1). Antibodies for flow cytometry anti-CD42a (GPIX, Xia.B4, M051-1), anti-CD42b (GPIb, Xia.G5, M040-1), anti-CD42c (GPIbβ, Xia.C3, M050-1), anti-CD42d (GPIbα, Gon.C2, M060-1), anti-CD61 (GPIIIa, integrin β3, Luc.H11-M031-1), anti-GPIIbIIIa JON/A-PE - M023-2, anti-GPVI (JAQ1)-M011-1, and anti-CD62P-FITC (M130-1) were obtained from Emfret analytics, Germany.<br>- Secondary antibodies for Western blot were from Li-Cor: https://www.licor.com/bio/reagents/irdye-secondary-antibodies
Validation	-Mfsd2b antibodies have been validated using Mfsd2b knockout sample (PMID:29045386). Tmem16F antibody was validated by provider laboratory (Professor Shigekazu Nagata, PMID: 26417084). Commercial antibodies have been validated by manufacturer and literature.

Animals and other organisms

Policy information about studies involving animals; ARRIVE guidelines recommended for reporting animal research

Laboratory animals	Mfsd2b knockout mice have been generated in our previous studies (PMID:29045386). EpoR-GFP-cre mice were generated previously (PMID:15090451); PF4-Cre mice were generated previously (PMID: 17032923).
Wild animals	NA
Field-collected samples	NA
Ethics oversight	Mice were maintained in normal chow diets. All experimental protocols were approved by IACUC committees under National University of Singapore.

Note that full information on the approval of the study protocol must also be provided in the manuscript.

Flow Cytometry

Plots

Confirm that:

☒ The axis labels state the marker and fluorochrome used (e.g. CD4-FITC).

☒ The axis scales are clearly visible. Include numbers along axes only for bottom left plot of group (a 'group' is an analysis of identical markers).

☒ All plots are contour plots with outliers or pseudocolor plots.

☐ A numerical value for number of cells or percentage (with statistics) is provided.

Methodology

Sample preparation	Blood and platelet samples were freshly collected from mice. Samples were processed as described in the method sections. Non-fixed cells were used for flow cytometry analysis immediately.
Instrument	BD LSR fortessa was used for flow cytometry analysis
Software	FlowJo was used for analyzed of flow cytometry data
Cell population abundance	Platelets were enriched in platelet rich plasma (PRP) fractions. During flow cytometry, platelets are acquired by FSC/SSC and detected by CD41 antibody. Erythrocytes were excluded by staining with Ter119. More than 98-99% platelets were enriched in PRP.
Gating strategy	Due to small cell size, platelets were first gated using FSC/SSC and are defined as CD41+Ter119- population. CD41+Ter119- platelets were selected for JON/A and CD62P expression in the resting and thrombin-activated experiments. For cell surface marker (CD42a, CD42b, CD42c, CD42d, CD61, and GPVI) determination, PRR was used. Similar gating strategy was uised. Mean Fluorescence Intensity (MFI) was calculated for JON/A, CD62P, and CD42a, CD42b, CD42c, CD42d, CD61, and GPVI in FlowJo.

☒ Tick this box to confirm that a figure exemplifying the gating strategy is provided in the Supplementary Information.
